# Deep Eutectic Solvents as Media for the Prebiotic DNA-Templated Synthesis of Peptides

**DOI:** 10.3389/fchem.2020.00041

**Published:** 2020-01-31

**Authors:** Samuel Núñez-Pertíñez, Thomas R. Wilks

**Affiliations:** School of Chemistry, University of Birmingham, Birmingham, United Kingdom

**Keywords:** prebiotic, translation, deep eutectic solvent, templated synthesis, nucleic acids

## Abstract

Translation of genetic information into peptide products is one of the fundamental processes of biology. How this occurred prebiotically, in the absence of enzyme catalysts, is an intriguing question. Nucleic acid-templated synthesis (NATS) promotes reactions by bringing building blocks tethered to complementary DNA strands into close proximity and has been shown to enable peptide synthesis without enzymes—it could therefore serve as a model for prebiotic translation of information stored in nucleic acid sequences into functional peptides. The decomposition of highly reactive DNA adapters has so far limited the effectiveness of NATS, but these studies have been performed exclusively in aqueous solution. Deep eutectic solvents (DESs) have been proposed as a feasible solvent for prebiotic replication of nucleic acids, and here are studied as media for prebiotic translation using NATS as a model. DESs are shown to enhance the stability of DNA-conjugated activated esters, the precursors of peptides. However, this enhanced stability was coupled with decreased amine reactivity that hampered the formation of peptide bonds in DESs. These properties are exploited to demonstrate the storage of DNA-conjugated activated esters in a DES followed by transfer into aqueous buffer to activate the NATS of peptides “on demand.” These findings, together with the reported functions of DESs in prebiotic processes, shed light on how DESs could have facilitated the non-enzymatic translation of genetic information into functional peptides on the early Earth.

## Introduction

Living organisms preserve the information required to synthesize their molecular components in nucleic acid sequences (Crick, 1970). The replication of this genetic material, and its translation into active catalytic proteins is regulated and catalyzed by highly complex molecular machines. Perhaps the most impressive example is the biosynthesis of peptides and proteins by the ribosome (Steitz, 2008; Yonath, 2009). A key question is how these processes were performed prebiotically, in the absence of such complex machinery. Based on the fact that the core of the ribosome is made from RNA, it has been proposed that replication and translation were first performed by simple nucleic acids, giving rise to the “RNA world” (Crick, 1968; Orgel, 1968; Kruger et al., 1982; Guerrier-Takada et al., 1983; Gilbert, 1986) and “RNA/peptide world” hypotheses (Yarus, 2001; Turk et al., 2010). Several nucleic acid systems that display enzyme-free self-replication have been described (Ekland and Bartel, 1996; Johnston et al., 2001; Paul and Joyce, 2002; Li et al., 2017; Hänle and Richert, 2018; Liu et al., 2018; Mariani et al., 2018; Zhang et al., 2019), and there have been a handful of mechanisms proposed for prebiotic translation (Zhang and Cech, 1997; Tamura and Schimmel, 2001, 2004). However, these studies have highlighted a number of obstacles to achieving efficient replication and translation in the absence of enzymes: (1) the hydrolysis of the highly reactive, activated monomers required for both processes is rapid in aqueous solution (Kanavarioti et al., 1989); (2) hydrolysed monomers can inhibit replication and induce errors in translation (Deck et al., 2011); (3) after replication, long strands will hybridize to form stable duplexes, blocking further replication by template inhibition (Szostak, 2012).

Most studies in this area have assumed that the solvent for these prebiotic processes was aqueous, but deep eutectic solvents (DESs) have recently emerged as an alternative (He et al., 2017). DESs are a family of solvents, closely related to ionic liquids, composed of a hydrogen bond donor and a salt that form a eutectic mixture at a specific molar ratio (Abbott et al., 2003). Most DESs have high viscosity, low vapor pressure and a very high concentration of ionic species (Zhang et al., 2012; Smith et al., 2014), and their basic components, such as glycerol or urea, are likely to have been present on the early Earth (Kaiser et al., 2015; Okamura et al., 2019). DESs composed of quaternary ammonium salts and hydrogen bond donors (e.g., reline, a mixture of urea and choline chloride) have been shown to be compatible with protein and nucleic acid biomolecules (Wagle et al., 2014; Xu et al., 2017; Pätzold et al., 2019). For example, it was demonstrated that DNA was stable and retained its structural integrity for at least 6 months when stored in a DES (Mondal et al., 2013), that tertiary structures such as A- and B-DNA duplexes, and G-quadruplexes, were maintained (Mamajanov et al., 2010; Lannan et al., 2012; Zhao et al., 2013; Gállego et al., 2015), and that enzymatic peptide synthesis could be performed efficiently (Maugeri et al., 2013). DESs have also been shown to be favorable media for nucleic acid replication. For example, DESs promote the formation of activated organophosphates from mineral phosphate, which is a necessary step in the formation of the activated monomers required for replication (Gull et al., 2014, 2017; Burcar et al., 2016). The high viscosities of DESs also enable more efficient non-enzymatic replication: the faster diffusion of monomers compared to long oligonucleotides in these solvents has been shown to help overcome the template inhibition problem (He et al., 2017). High viscosity also favors intramolecular folding over intermolecular interactions, enabling, for example, the recovery of ribozyme activity following a replication process (He et al., 2019).

Based on this previous work around replication, we asked whether DESs could also be suitable media for non-enzymatic translation and chose nucleic acid-templated synthesis (NATS) of a dipeptide as a simple model for this process. NATS promotes reactions by using hybridization to bring reactants tethered to complementary oligonucleotides into close proximity, and has been extensively used in the literature for the programmed production of oligopeptides (Li and Liu, 2004; O'Reilly et al., 2017). Importantly, this previous work has been limited by hydrolysis of the activated building blocks required (Meng et al., 2016). Here, we investigate the NATS of peptides in an archetypal DES, glycholine (a mixture of glycerol and choline chloride). We find that glycholine protects DNA-conjugated activated esters, the necessary precursors of oligopeptide products, from solvolysis. By comparing the NATS of peptides in DESs and aqueous buffered solution we also uncover the inhibitory effect of glycholine on amide bond formation. We exploit these properties to demonstrate the extended storage of otherwise unstable peptide building blocks in glycholine, followed by shuttling into aqueous solution to activate NATS “on demand.” Combined with recent theories about the role of compartmentalisation in prebiotic self-replicating systems (Mann, 2012), these experiments hint at a possible role for DESs in enabling non-enzymatic construction of oligopeptides on the early Earth.

## Materials and Methods

*Nuclear magnetic resonance (NMR):* One-dimensional ^1^H and ^13^C NMR, and two-dimensional NMR spectra were recorded on a Bruker Advance 300 MHz, Bruker Advance III 400 MHz or a Bruker Advance III 500 MHz instrument at 25°C. Deuterated solvents chloroform-*d* (99.8 D atom%), methanol-*d*_4_ (99.8 D atom%), dimethyl sulfoxide-*d*_6_ (DMSO-*d*_6_, 99.8 D atom%) acetone-*d*_6_ (99.9 D atom%) were purchased from Sigma Aldrich. The residual non-deuterated solvent peak was used as a chemical shift (δ, ppm) internal standard. The data were processed using Mestrenova (Mestrelab research) v.12.0.2 and ADC/NMR software. *High resolution mass spectrometry (HRMS):* HRMS was performed on a Bruker Q-ToF Maxis Plus spectrometer or on a Waters Xevo GS2-XS qToF system. A relative error under 5 ppm was ensured. *Reversed-phase high performance liquid chromatography (RP-HPLC):* RP-HPLC was performed in a Varian 920LC system with a photodiode array UV detector (PDA), and a fluorescence detector. *Liquid chromatography-Mass spectrometry (LC-MS):* LC-MS was performed on an Agilent 1200 HPLC system coupled to a Bruker AmazonX high resolution ion trap, in negative ion mode. The desalted oligonucleotide samples were eluted though a XBridge oligonucleotide BEH C18 column (130 Å, 2.5 μm, 4.6 × 50 mm) using a 5 vol% MeOH, 10 mM ammonium acetate (buffer A) and a 70 vol% MeOH, 10 mM ammonium acetate (buffer B) solvent system. The data were processed using Compass Data Analysis (Bruker) v.4.1 software, and the MaxEnt integrated deconvolution algorithm. Alternatively, LC-MS was performed on a Waters ACQUITY UPLC system coupled to a Xevo GS2-XS qToF mass spectrometer in negative sensitivity mode with leucine-enkephalin [M–H]^−^ 554.2620 Lockspray. The oligonucleotides were eluted through an AQUITY UPLC oligonucleotide BEH C18 column (130 Å, 1.7 μm, 2.1 × 50 mm) using a 50 mM triethylammonium acetate (TEAA, pH 7.0) solution in H_2_O (buffer A) and a 50 mM TEAA solution in MeCN (buffer B) at 60°C and a 0.2 mL·min^−1^ flow. *Fluorescence spectroscopy:* Fluorescence spectral data were recorded on an Agilent Cary Eclipse fluorescence spectrophotometer equipped with a photomultiplier tube (PMT) detector. Quartz cuvettes from Starna scientific (Type 3/Q/10) with four polished sides were used for fluorescence. The emission and excitation spectra were recorded using Cary Eclipse v.1.2.0.0 software. *Karl Fischer titration:* The water content in DESs was determined using an automated CA-200 (Mitsubishi chemicals) Karl Fischer coulometric titrator. Aqualine (Fisher) auxiliary reagent mixture and Aquamicron solution P (Aquamicron, 3.8–4.0 mg H_2_O·mL^−1^) calibration standards were used. *pH measurements:* An Accumet AP110 pH meter kit (Fisher) equipped with a pH sensitive glass electrode was used to determine the pH in aqueous solution. Prior to sample analysis, the instrument was calibrated with pH 4, pH 7, and pH 10 standard solution buffers.

### DNA Sequences and Modifications

Sequences were optimized, and the expected assemblies predicted with NUPACK (Zadeh et al., 2009). Modified DNA strands were purchased from Integrated DNA Technologies and further modified as described below. Sequence data and modification structures are given in Table S1 and Scheme S1.

### Synthesis of DESs

DESs were prepared by mixing appropriate amounts of hydrogen bond donor and choline chloride in a Schlenk round bottom flask under positive nitrogen pressure, heating to 100°C and stirring until a homogeneous mixture was obtained. DESs were dried at 80°C under vacuum prior to use (Abbott et al., 2004). The H_2_O content of glycholine was determined by Karl Fischer titration. H_2_O content was then adjusted to the desired value. For simplicity, we refer to the volume percentage content of H_2_O in the DES using a subscript number e.g., glycholine containing 7 vol% H_2_O is written as Gly_0.07_.

### Measurement of DES Solution pH

DES (1 mL) was diluted in H_2_O (4 mL) and the pH was determined using a pH-sensitive glass electrode. The error was evaluated in two different series of samples, by performing triplicate measurements and determining the standard deviation.

### ^1^H-NMR Spectroscopy Study of The Stability of Small Molecule Activated Esters

Solutions of **1** (5 mM) in different solvent mixtures were stirred at 24 °C. After 2, 4, 6 and 24 h the solutions were extracted with 0.7 mL of either acetone-*d*_6_ (DES solutions) or chloroform-*d* (phosphate buffer solutions). The relative proportions of **1** and **3** were determined by ^1^H-NMR spectroscopy (Figure S1).

### RP-HPLC Stability Study of Activated Esters

Activated ester **1** (4.1 mg) was dissolved in DES (2 mL). Each solution was prepared 40 min after the previous one. The solutions were stirred over a period of 29 h. After stirring for 5, 24, and 29 h, a 100 μL aliquot of each sample was diluted in H_2_O (900 μL) and the sample analyzed by RP-HPLC. Method: column Discovery C18 (Sigma Aldrich, 5 μm, 10 × 4.6 cm), flow 1 mL·min^−1^, temperature 40°C and injection volume 10 μL. Solvent A: H_2_O, 0.05 vol% TFA. Solvent B: 70 vol% MeCN, 0.05 vol% TFA.

### Thermal Stability of dsDNA in DESs

The thermal stability of dsDNA in DES solutions was determined using fluorophore and quencher labeled DNA strands (**S15** and **S16**). Solutions of 100 nM dsDNA were briefly centrifuged and placed in a qPCR instrument. Samples were heated and cooled at 1°C·min^−1^. The melting temperature (*T*_m_) was determined as the middle-point of the sigmoidal trace defined by the fluorescence emission intensity as a function of temperature.

### Synthesis of NHS-Activated Fluorescein, S18 (Gao et al., 2002)

**Scheme 1:**
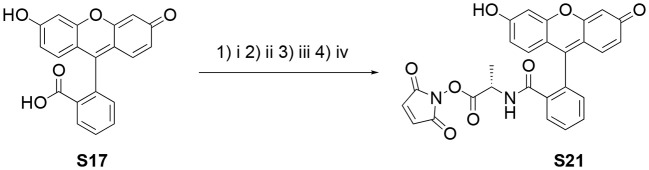
Synthesis of the *N*-hydroxymaleimide ester **S21**. (i) SuOH, DCC, DMF, 60°C, 2 h; (ii) TsOH·H-Ala-OBn, Et_3_N, DCM, r.t. 3 h; (iii) Pd/C (10 wt%), H_2_, MeOH, r.t., 1.5 h; (iv) *N*-hydroxymaleimide, DCC, EtOAc:1,4-dioxane (2:1), r.t., o.n.

Fluorescein free carboxylic acid (**S17**, 3.322 g, 10 mmol) and *N-*hydroxysuccinimide (1.151 g, 10 mmol) were dissolved in dry DMF (19 mL) under positive nitrogen pressure in a dry Schlenk tube (Scheme 1). Then, *N,N*′*-*dicyclohexylcarbodiimide (DCC, 2.063 g, 10 mmol) was added and the resulting solution stirred at 60°C for 2 h under positive nitrogen pressure. *N,N*′-dicyclohexylurea (DCU) was removed by cooling down the reaction mixture to −20°C for 2 h and the white precipitate was filtered off. The solvent was removed under reduced pressure and the crude solid was purified by SiO_2_ column chromatography using an EtOAc/acetone gradient. The desired fractions were combined to produce **S18** as a bright orange solid (2.002 g, 47%). The ^1^H-NMR spectroscopic analysis was in good agreement with the reported characterization (Gao et al., 2002). **TLC** (EtOAc:MeOH 6:4) R_f_ = 0.1. ^**1**^**H-NMR** (300 MHz, DMSO-*d*_6_) δ (ppm): 8.36 (1H, d *J* = 7.7 Hz, Ar-*H*), 7.96 (2H, m, Ar-*H*), 7.65 (1H, d *J* = 7.5 Hz, Ar-*H*), 6.79 (2H, d *J* = 9.6 Hz, Ar-*H*), 6.53 (4H, m, Ar-*H*), 2.73 (4H, s^br^, (C*H*_2_)_2_ succinimide). **HRMS** (ESI–) *m/z* [M–H]^−^ calcd. 428.0770 found 428.0763.

### Synthesis of S19

**S18** (1.000 g, 2.23 mmol) and TsOH·H-*L*-Ala-OBn (1.637 g) were dissolved in dry DMF (15 mL) under positive nitrogen pressure in a dry round bottom Schlenk flask. Then, Et_3_N (1.1 mL, 8.16 mmol) was added drop wise over 10 min and the resulting solution was stirred at room temperature for 6 h. The solvent was removed under reduced pressure and the crude product was purified by SiO_2_ column chromatography using a DCM/MeOH (100:0 to 75:15) gradient to produce **S19** as a bright orange solid (704 mg, 61%). **TLC** (EtOAc:MeOH 6:4) R_f_ = 0.6. ^**1**^**H-NMR** (400 MHz, methanol-*d*_4_) δ (ppm): 7.76 (1H, m, Ar-*H*), 7.55 (1H, m, Ar-*H*), 7.41 (1H, m, Ar-*H*), 7.21 (2H, m, Ar-*H*), 7.11 (4H, m, Ar-*H*), 6.88 (1H, m, Ar-*H*), 6.56 (1H, m, Ar-*H*), 6.51 (1H, m, Ar-*H*), 6.45 (1H, m, Ar-*H*), 6.20 (1H, d *J* = 8.7 Hz, Ar-*H*), 5.97 (1H, dd *J*_1_ = 8.7 Hz *J*_2_ = 2.5 Hz, Ar-*H*), 4.80 (2H, m, OC*H*_2_), 3.59 (1H, q *J* = 7.2 C*H*-CH_3_), 1.02 (3H, d *J* = 7.2 Hz, CH-C*H*_3_). ^**13**^**C-NMR** (100 MHz, methanol-*d*_4_) δ (ppm): 171.75 (*C*=O), 168.79 (*C*=O), 160.29 (*C*=O), 160.13 (Ar*C*), 154.54 (Ar*C*), 154.36 (Ar*C*), 154.24 (Ar*C*), 136.95 (Ar*C*), 134.40 (Ar*C*-H), 132.13 (Ar*C*-H), 130.71 (Ar*C*-H), 130.60 (Ar*C*-H), 129.89 (Ar*C*-H), 129.53 (Ar*C*), 129.37 (Ar*C*-H), 129.35 (Ar*C*-H), 129.15 (Ar*C*), 129.09 (Ar*C*), 125.13 (Ar*C*-H), 123.55 (Ar*C*-H), 113.26 (Ar*C*-H), 112.95 (Ar*C*-H), 110.26 (Ar*C*), 109.24 (Ar*C*), 103.59 (Ar*C*-H), 103.08 (Ar*C*-H), 68.08 (O*C*H_2_), 51.55 (N*C*H), 15.44 CH_3_). **HRMS** (ESI–) *m/z* [M–H]^−^ calcd. 492.1447 found 492.1457.

### Synthesis of S20

Pd/charcoal (50 mg, 10 wt%) was placed in a dry round bottom Schlenk flask under positive nitrogen pressure. **S19** (500 mg, 1.01 mmol) was added and dissolved in degassed MeOH (50 mL). The nitrogen atmosphere was replaced with hydrogen by bubbling the solution for 3 min with a hydrogen balloon. The positive hydrogen pressure was maintained for 1.5 h with constant stirring at room temperature. The solid-supported catalyst was removed by filtration through celite. The solvent was removed under reduced pressure to produce **S20** as a bright yellow solid (375 mg, 92%). **TLC** (DCM:MeOH 9:1) R_f_ = 0.2. ^**1**^**H-NMR** (500 MHz, methanol-*d*_4_) δ (ppm): 7.05 (1H, m, Ar-*H*), 6.61 (3H, m, Ar-*H*), 6.48 (2H, m, Ar-*H*), 6.41 (1H, dd *J*_1_ = 8.7 Hz *J*_2_ = 2.4 Hz, Ar-*H*), 3.67 (1H, q *J* = 7.2 Hz, C*H*CH_3_), 1.20 (3H, d *J* = 7.2 Hz CHC*H*_3_). ^**13**^**C-NMR** (160 MHz, methanol-*d*_4_) δ (ppm): 173.6 (*C*=O), 169.1 (*C*=O), 160.6 (*C*=O), 160.4 (Ar*C*), 154.9 (Ar*C*), 154.6 (Ar*C*),154.4 (Ar*C*), 134.5 (Ar*C*), 132.5 (Ar*C*), 132.4 (Ar*C*-H), 130.9 (Ar*C*-H), 130.0 (Ar*C*-H), 113.4 (Ar*C*-H), 113.0 (Ar*C*-H), 110.6 (Ar*C*), 109.6 (Ar*C*), 103.7 (Ar*C*-H), 103.2 (Ar*C*-H), 51.7 (*C*H-CH_3_), 15.7 (CH-*C*H_3_). **HRMS** (ESI–) *m/z* [M–H]^−^ calcd. 402.0983 found 402.0982.

### Synthesis of S21

**S20** (50 mg, 0.124 mmol), DCC (25 mg, 0.121 mmol) and *N*-hydroxymaleimide (14 mg, 0.124 mmol) were dissolved in EtOAc/1,4-dioxane (2:1, 2 mL) and stirred at room temperature overnight. Then, the solvent was lyophilised on a Schlenk line, the crude solid was dissolved in EtOAc (2.5 mL) and cooled down to 4°C for 1 h. The white precipitate of DCU was removed by centrifugation at 4°C and 21 kRCF for 15 min. The supernatant was dried to produce **S21** as a bright yellow powder (58 mg, 95%). The NMR spectra showed extra peaks which were attributed to atropoisomers and the presence of trace amounts of DCU. ^**1**^**H-NMR** (500 MHz, DMSO-*d*_6_) δ (ppm): 9.99 (1H, s, O*H* or N*H*), 9.89 (1H, s, O*H* or N*H*), 7.83 (1H, m, Ar-*H*), 7.57 (2H, m, Ar-*H*), 7.17 (2H, s^br^, maleimide Ar-*H*), 7.05 (1H, m, Ar-*H*), 6.61 (2H, m, Ar-*H*), 6.55 (1H, m, Ar-*H*), 6.49 (2H, m, Ar-*H*), 6.38 (1H, m, dd *J*_1_ = 8.7 Hz *J*_2_ = 2.4 Hz, Ar-*H*), 4.11 (1H, q *J* = 7.1 Hz, C*H*-CH_3_), 1.26 (3H, d *J* = 7.1 Hz, CH-C*H*_3_)0.1^**3**^**C-NMR** (160 MHz, DMSO-*d*_6_) δ (ppm): 167.28 (*C*=O), 165.96 (*C*=O), 164.38 (*C*=O), 158.71 (*C*=O), 157.36 (Ar*C*), 153.20 (Ar*C*), 152.13 (Ar*C*), 133.43 and 133.09 (maleimide *C*H=*C*H), 131.91 (Ar*C*-H), 130.07 (Ar*C*-H), 129.68 (Ar*C*), 129.45 (Ar*C*-H), 128.78 (Ar*C*-H), 123.85 (Ar*C*-H), 122.58 (Ar*C*-H), 112.37 (Ar*C*-H), 112.11 (Ar*C*-H), 108.47 (Ar*C*), 107.62 (Ar*C*), 102.41 (Ar*C*-H), 102.06 (Ar*C*-H), 48.16 (*C*H-CH_3_), 15.05 (CH-*C*H_3_). **HRMS** (ESI–) *m/z* [M–H]^−^ calcd. 497.0985 found 497.0992.

### Fluorescence Spectroscopy Study of the Stability of DNA-Conjugated Activated Esters

The tetramethylrhodamine (TAMRA)/disulfide-labeled DNA hairpin **S4** (1 μL, 1 mM in H_2_O, 1 nmol) was thermally annealed with the complementary biotinylated strand **S5** (1.1 μL, 1.1 mM in H_2_O, 1.1 nmol) in phosphate buffer solution (PBS, 10 μL, 0.1 M, pH 7.0). The disulfide group was reduced with tris(2-carboxyethyl)phosphine hydrochloride (TCEP, 0.5 μL, 0.5 M in H_2_O) at 18°C for 1.25 h. Then, the resulting double stranded DNA (dsDNA) was conjugated to streptavidin-coated magnetic nanoparticles (Dynabeads, Thermofisher), and transferred into Gly_0.07_ (200 μL). The maleimide ester **S21** (1 mg, 2.5 μmol) was added to the solution and allowed to react for 2 h. The nanoparticle-supported DNA-conjugated activated ester was transferred into clean Gly_0.07_ and the hairpin was released by toehold-mediated strand exchange by addition of one equivalent of strand **S6** at 18°C for 2 h to produce an approximately 5 μM solution of **4**. Finally, the solution of **4** (5 μL) was diluted in the DES of interest (95 μL), the resulting solution briefly centrifuged, and the fluorescein (FAM) fluorescence recorded over 24 h in a Mx3005P qPCR instrument (Agilent). The spectral overlap between FAM fluorophore (**S21)** and TAMRA-labeled DNA (**S4**) was also determined (Figure S4).

### DNA-Templated Synthesis of Peptide Bonds

Disulfide-labeled DNA **S7** (10 μL, 1 mM in H_2_O, 10 nmol) was mixed with TCEP solution (2 μL, pH 4 corrected with 5 M NaOH, 1 μmol) in a microcentrifuge tube. The solution was shaken at 18°C for 1.25 h. Maleimide activated ester **S21** (108 μL, 37 mM in DMF, 4 μmol) was added and the combined solution shaken at 18°C for 1.0 h to produce the DNA-conjugated activated ester, **6**. The volume of the solution was reduced to approximately 50 μL by freeze-drying. The previous solution was diluted with Gly_0.07_ (4,950 μL), and amine-labeled DNA **7** (10 μL, 1 mM in H_2_O, 10 nmol) added. The resulting mixture was vigorously shaken until the solution was homogeneous and then shaken at 18°C overnight. The DES solution was diluted with an excess of H_2_O and the DNA strands transferred into H_2_O for HPLC/LC-MS analysis by repeated centrifugal ultrafiltration using a 3 kDa molecular weight cut-off (MWCO) Amicon spin filter. When the DNA-templated reaction was performed in aqueous solution, the activated ester solution in DMF was diluted with PBS (4,950 μL, 0.1 M, pH 7.5) containing the complementary amine-labeled DNA **7**.

### Amine-Labeled DNA Reactivity in DES/Triethylamine (Et_3_N)

Amine-labeled DNA **10** (1 μL, 1 mM in H_2_O, 1 nmol) was diluted in the appropriate solvent (100 μL). *N*-hydroxysuccinimide ester **11** (~0.3 mg, 1 μmol) was added, and the mixture shaken overnight at 20°C. The product was transferred into H_2_O by dilution of the samples and successive centrifugal ultrafiltration using a 3 kDa MWCO Amicon spin filter. The product was analyzed by HPLC and LC-MS.

### Storage of Activated Esters in Gly_0.07_

Disulfide-labeled DNA **S11** (3 μL, 1 mM in H_2_O, 3 nmol) was mixed with TCEP solution (3 μL, pH 4 corrected with 5 M NaOH, 1.5 μmol) in a microcentrifuge tube. The solution was shaken at 18°C for 1.25 h. Maleimide activated ester **S21** (54 μL, 22 mM in DMF, 1.2 μmol) was added and the combined solution was shaken at 18°C for 1.0 h to produce the DNA-conjugated activated ester **13**. This sample was divided into 3 aliquots and used as follows. (1) *Storage in Gly*_0.07_: the activated ester solution (20 μL) was diluted with Gly_0.07_ (180 μL), and the resulting solution was shaken at 18°C for 2.0 h. The solution was diluted with PBS (1.3 mL, 0.1 M, pH 7.5) containing complementary amine-labeled DNA **15** (1 μL, 1 mM in H_2_O, 1 nmol). The solution was shaken at 18°C for 5 h. Finally, the solution was diluted with 1 vol. of H_2_O, the precipitate was centrifuged, and the DNA products were transferred into H_2_O by successive steps of dilution of the supernatant with H_2_O and concentration by centrifugal ultrafiltration on a 3 kDa MWCO Amicon spin filter. The product was analyzed by HPLC and LC-MS. (2) *Storage in PBS*: the activated ester solution (20 μL) was diluted with PBS (180 μL, 0.1 M, pH 7.0), and the resulting solution was shaken at 18°C for 2.0 h. Then, the sample was treated as in (1). (3) *Fast reaction in PBS:* the activated ester solution (20 μL) was diluted with PBS (1,480 μL, 0.1 M, pH 7.5) containing complementary amine-labeled DNA **15** (1 nmol). The solution was shaken at 18°C for 5.0 h. The products were transferred into H_2_O for LC-MS analysis as described previously. (4) *Off-template control:* maleimide activated ester (**S21**) (18 μL, 22 mM in DMF, 400 nmol) was diluted in PBS (1,482 μL, 0.1 M, pH 7.5) containing amine-labeled DNA **15** (1 μL, 1 mM in H_2_O, 1 nmol). The solution was shaken at 18°C for 5.0 h and the product was transferred into H_2_O for LC-MS analysis as described above.

## Results and Discussion

### The Stability of Activated Esters in DES and Aqueous Buffer

**Figure 1:**
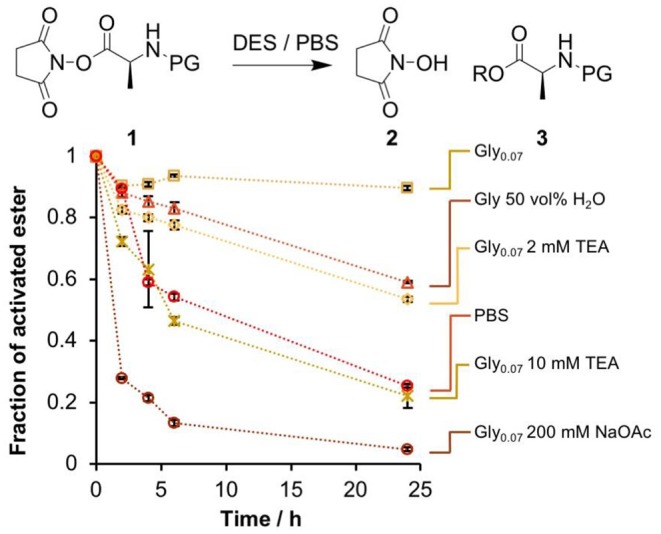
Comparison of the stability of the NHS ester 1 in several glycholine and aqueous PBS solutions. The fraction of 1 remaining at set time points was determined by ^1^H-NMR spectroscopy by extraction into an immiscible deuterated solvent. Error bars correspond to the standard deviation over multiple integration events.

We used the *N*-hydroxysuccinimide ester of *L*-alanine (NHS-Ala) as a model activated peptide building block. This had similar solution stability to more prebiotically feasible activating groups while allowing us to use established techniques to prepare the required DNA-conjugated esters (He and Liu, 2011). A preliminary stability experiment was performed using a small-molecule NHS-Ala, **1**. We followed the hydrolysis of **1** in several glycholine/water mixtures and in aqueous PBS using proton nuclear magnetic resonance (^1^H-NMR) spectroscopy (Figure 1 and Figure S1). **1** was dissolved in the appropriate solvent mixture and then left for 24 h at 24°C. Samples were extracted with immiscible deuterated solvent at set timepoints, and the relative proportions of activated ester and degradation products (**2** and **3**) were determined based on their distinctive methyl ^1^H-NMR signals. Both the proportion of water and the presence of basic additives were investigated for their effect on the rate of ester degradation.

As expected, rapid degradation of the ester in aqueous PBS was observed, with only 25% remaining after 24 h. In stark contrast, degradation in Gly_0.07_ was very slow, with ~90% of the activated ester intact after 24 h. Increasing the H_2_O content to 50 vol% accelerated solvolysis substantially. We attempted to deliberately accelerate the degradation in glycholine by use of a basic additive, triethylamine (Et_3_N). The presence of 2 mM Et_3_N in Gly_0.07_ had the same destabilizing effect as increasing the water content to 50 vol%, while 10 mM Et_3_N led to a similar rate of degradation to that observed in PBS. The effect of basic additives was studied by diluting the DES in H_2_O and measuring the pH of the resulting solution. 2 mM Et_3_N produced a pH of 7.5, while 10 mM Et_3_N produced a pH of 9.9 (Table S2), it therefore seems likely that the observed differences in degradation rate arose as a result of the increased basicity of the solution.

The above ^1^H-NMR analysis could have been biased by differences in the extraction efficiency of **1** and **3** into the deuterated solvent used, so a complementary study that did not rely on this assumption was performed using reversed-phase high performance liquid chromatography (RP-HPLC, Figure S2). The results were highly consistent with the ^1^H-NMR study, so we concluded that glycholine was indeed effective at stabilizing activated esters, and moved on to DNA-based experiments.

The thermal stability of double-stranded DNA (dsDNA) in glycholine/H_2_O was assessed by fluorescence quenching and revealed that the duplex remained stable down to at least 7 vol% H_2_O: in Gly_0.07_ the *T*_m_ of a 24 base pair dsDNA was 48°C (Figure S3). The addition of moderate volumes of H_2_O to DES has been used to reduce its viscosity and melting point (Ma et al., 2018; Smith et al., 2019), and resulted in increased conversion in enzymatic reactions (Durand et al., 2013; Guajardo et al., 2017). Furthermore, it has been shown that the microstructure of the DES is at least partially preserved upon the addition H_2_O, up to 50 wt% (Hammond et al., 2017; Gabriele et al., 2019). Given this precedent, as well as the good stability of activated esters demonstrated above, Gly_0.07_ was used for experiments from this point onwards.

**Figure 2:**
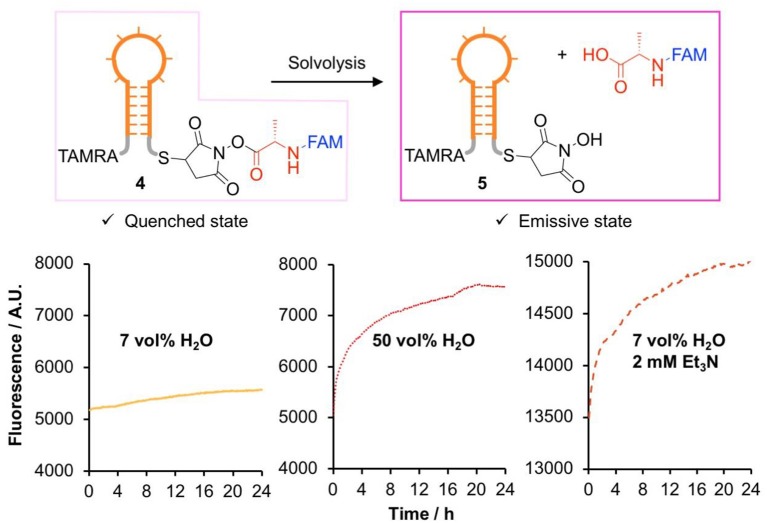
Comparison of the stability of DNA-conjugated *N*-hydroxysuccinimide esters in several glycholine mixtures studied by fluorescence dequenching.

We were interested in assessing the stability of DNA-conjugated activated esters *in situ*, avoiding the possibility of degradation occurring during analysis. To this end, a fluorescence quenching experiment was designed (Figure 2). A FAM-labeled NHS-Ala (**S21**) was synthesized from **S17** through a multi-step procedure (Scheme 1 and Scheme S2, intermediates **S18** to **S20**) and conjugated to a TAMRA-labeled DNA hairpin through thia-Michael addition. This design meant that in the ester form (**4**), FAM fluorescence would be quenched by the neighboring TAMRA group. Upon solvolysis of the ester to give **5**, the FAM-labeled amino acid would diffuse away, triggering a measurable increase in fluorescence. We measured the fluorescence excitation and emission spectra of FAM and TAMRA in glycholine to ensure good spectral overlap was maintained in this solvent (Figure S4).

The DNA hairpin was supported on streptavidin-coated magnetic nanoparticles and transferred to Gly_0.07_ for activated ester synthesis, ensuring minimal hydrolysis of the activated ester. Excess reagents were removed by magnetic purification, the hairpins were released into solution by toehold-mediated strand displacement and the nanoparticles removed with a magnet. The hairpin solution was diluted into an excess of the solvent of interest, and the evolution of the fluorescence over a 24 h period was recorded (Figure S5).

All experiments showed the expected increase in fluorescence due to solvolysis of the activated ester (Figure 2). While a rigorous quantitative kinetic analysis of the data was not possible due to uncertainties in the minimum and maximum fluorescence values, the results showed exactly the same trend as the small molecule experiments, with slow hydrolysis observed in Gly_0.07_. We therefore concluded that glycholine was also effective at stabilizing DNA-conjugated activated esters.

### Nucleic Acid-Templated Synthesis of Peptide Bonds in DES

**Figure 3:**
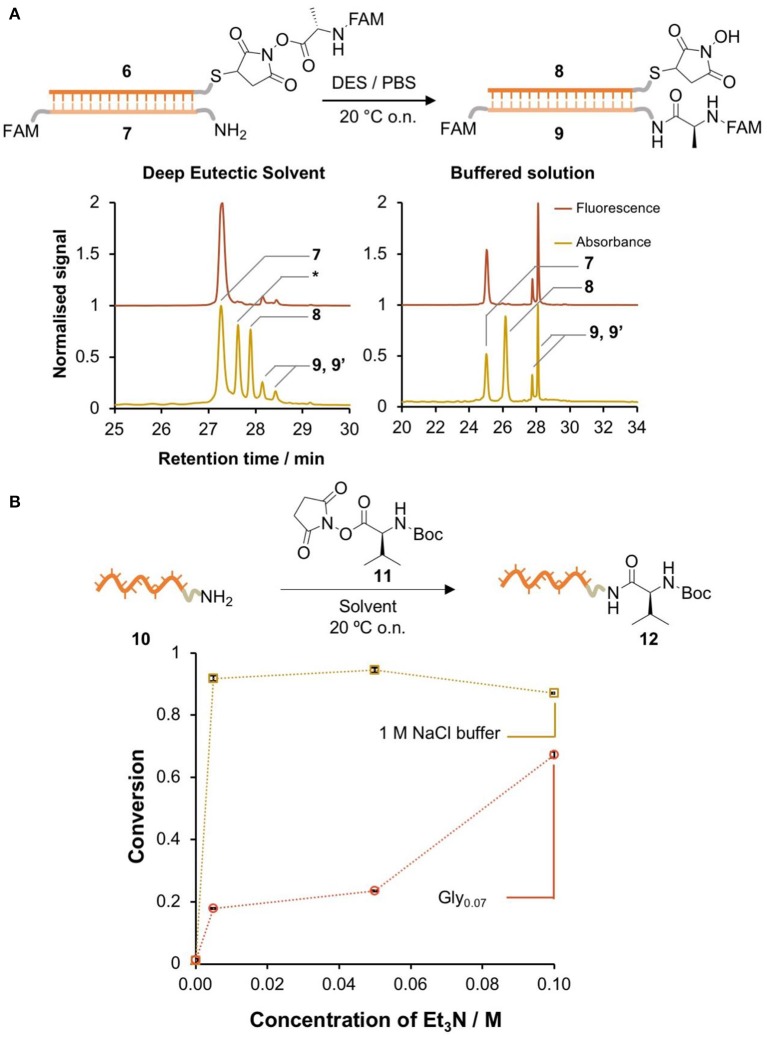
DNA-templated formation of peptide bonds in DES. **(A)** Single-step DNA-templated synthesis of a peptide bond in glycholine and in PBS. The conversion was determined by HPLC integration of the signals corresponding to 7, 9, and 9' (isomers of the product). Peaks were identified by LC-MS (Bruker AmazonX): 7 [M-H]^−^*m/z* calcd. 9337.2 found 9336.0; ^*^DNA-SH [M-H]^−^*m/z* calcd. 10615.2 found 10599.6; 8 [M-H]^−^ *m/z* calcd. 10712.0 found 10712.4; 9 and 9' (isomers) [M-H]^−^ *m/z* calcd. 9723.6 **(B)** comparison of the reactivity of an amine-labeled DNA (10) in the presence of an excess of activated ester (11) in Gly_0.07_ / Et_3_N and 1 M NaCl (aq)/Et_3_N. The conversion was determined by HPLC integration of the signals corresponding to 10 and 12. Peaks were identified by LC-MS (Bruker AmazonX): 10 [M-H]^−^*m/z* calcd. 3897.7 found 3898.6; 11 [M-H]^−^ *m/z* calcd. 4096.8 found 4097.7. Error bars correspond to the standard deviation over multiple integration events.

Having demonstrated the higher stability of the activated esters in glycholine, we moved to a single-step NATS reaction using NHS-Ala (Figure 3A). The DNA-conjugated activated ester **6** was prepared in 90 vol% DMF and then transferred into a DES solution containing the complementary acceptor strand **7**. The DMF content was reduced to 1 vol%, which did not substantially affect the duplex *T*_m_. Alternatively, **6** was synthesized in Gly_0.07_; this required longer reaction times (2 h) to reach the same yield attained in DMF, which we speculate was due to the higher viscosity of the DES. Controls mixing the acceptor strand with the small molecule reagents showed that no off-template reactions took place.

A control NATS reaction in PBS was also performed. While hydrolysis of the activated esters in aqueous solution was very fast, if the DNA conjugate **6** was isolated rapidly and transferred to a solution that contained the complementary amine-labeled DNA **7**, the amide product **9** was formed in 48% conversion. By contrast, the formation of **9** in DES was only detected in very low yield (Figure 3A). A series of attempts to improve the conversion through the use of additives were not successful in raising the yield of **9** above ~5% (Table S3). We hypothesized that the low conversion in DES was due to a lack of reactivity of the amine moiety. To test this, amino-modified DNA **10** was reacted with a large excess of a small-molecule activated ester **11** in aqueous 1 M NaCl and glycholine solution that contained varying concentrations of Et_3_N (Figure 3B). In aqueous solution, the conversion in the absence of base was very low, however the addition of a minimal amount of Et_3_N resulted in nearly quantitative conversion. By contrast, in DES, very large amounts of Et_3_N were required to achieve only moderate conversion (60%). It is interesting to note that NATS of peptide bonds has previously been reported in pH 7.5 buffered solution, similar to the pH of the glycholine/Et_3_N solutions measured above, so pH cannot by itself explain the lack of amine reactivity in these solvent mixtures (He and Liu, 2010).

**Figure 4:**
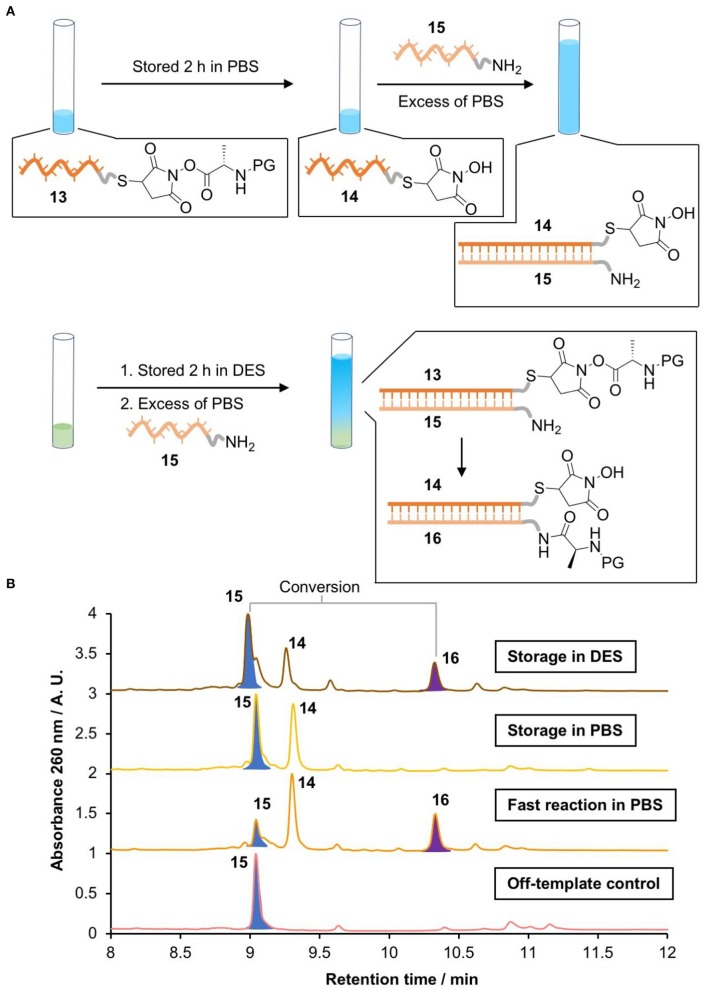
Activating NATS “on demand.” **(A)** Scheme of the storage of a DNA conjugated activated ester in PBS (0.1 M pH 7.0) or Gly_0.07_, followed by DNA-templated peptide bond formation in PBS. **(B)** LC-MS chromatograms of the products (Waters Xevo-G2-XS). 15 [M]^0^ *m/z* calcd. 10891.855 found 10891.522; 14 [M]^0^ *m/z* calcd. 9558.632 found 9558.581. 16 [M]^0^ *m/z* calcd. 11201.935 found 11201.489.

These results highlighted that glycholine by itself could not have promoted prebiotic peptide synthesis. Stabilization of the activated ester comes at the cost of reduced amine reactivity, and increasing the reactivity of the amine can only be achieved by use of additives that destabilize the ester. However, recent hypotheses have proposed an important role for compartmentalisation in prebiotic processes (Mann, 2012). One could imagine a prebiotic system in which activated species are generated and stored in pockets of DES, and then transferred into aqueous solution to facilitate the formation of peptide products. We designed a transfer experiment to investigate this possibility (Figure 4A).

The DNA-conjugated activated ester **13** was stored in PBS or glycholine for 2 h. The solution was then diluted with an excess of PBS containing the acceptor amino-DNA strand **15**. The templated synthesis of the peptide product **16** was then quantified via HPLC by comparing peak areas (Figure 4B). The identity of the product was confirmed by LC-MS. A positive control was performed by removing the storage step and adding the acceptor strand (**15**) immediately after the synthesis of the activated ester **13**. This resulted in 68% conversion to the peptide product. To check the importance of DNA templating, an off-template negative control was performed by adding the acceptor strand **15** to a solution of the small molecule activated ester **S21** at the same concentration as the DNA strand (670 nM). This showed no evidence of the formation of the peptide product **16**, as expected. When the DNA-templated peptide synthesis was attempted following storage of the activated ester in PBS for 2 h, no product was observed (Figure 4B). By contrast, DNA-templated synthesis following storage in glycholine resulted in 30% conversion to the peptide product.

## Discussion

Here, we demonstrate that glycholine-based DESs have a positive impact on the stability of DNA-conjugated activated esters when compared with buffered aqueous solutions. However, there is a marked reduction in the reactivity of amines, which prevents the NATS of peptides in these solvents. Our results highlight that it is very challenging to produce activated esters in aqueous media for subsequent templated reactions, as they quickly decompose; however, the synthesis of the activated ester in Gly_0.07_ provides a solution to this problem. By applying a two-stage process in which the activated ester was stored in DES and then transferred into PBS solution to trigger templated synthesis we achieved a moderate yield of 30%. By comparison, no product was observed if the activated ester was stored in PBS.

It may be possible to circumvent the issue of amine deactivation we observe by the use of alternative DESs or other viscous solvents. For example, glycerol containing an appropriate concentration of NaCl has been shown to stabilize DNA duplexes (Bonner and Klibanov, 2000). Future work could investigate whether such solvent mixtures are more effective at promoting nucleic acid-templated peptide synthesis, and thus shine further light on whether increased activated ester stability is inevitably linked to decreased amine reactivity in these environments.

Finally, it is interesting to speculate how our results might be relevant to prebiotic translation. The high viscosity of DESs means that they mix only slowly with aqueous solutions, so this may have allowed a primitive form of compartmentalization in which the production and stabilization of activated species took place in a DES phase, followed by diffusion into the aqueous phase where templated chemistry could occur. Future experiments using simple DES/aqueous interfaces of this kind could provide fascinating insights into such prebiotic processes.

## Data Availability Statement

The datasets generated for this study are available on request to the corresponding author.

## Author Contributions

SN-P and TW designed the experiments, analyzed the data, and wrote and formatted the manuscript. SN-P carried out the experimental work.

### Conflict of Interest

The authors declare that the research was conducted in the absence of any commercial or financial relationships that could be construed as a potential conflict of interest.

## References

[B1] Abbott A. P. Boothby D. Capper G. Davies D. L. Rasheed R. K. (2004). Deep eutectic solvents formed between choline chloride and carboxylic acids: versatile alternatives to ionic liquids. J. Am. Chem. Soc. 126, 9142–9147. 10.1021/ja048266j 15264850

[B2] Abbott A. P. Capper G. Davies D. L. Rasheed R. K. Tambyrajah V. (2003). Novel solvent properties of choline chloride/urea mixtures. Chem. Commun. 2003, 70–71. 10.1039/b210714g 12610970

[B3] Bonner G. Klibanov A. M. (2000). Structural stability of DNA in nonaqueous solvents. Biotechnol. Bioeng. 68, 339–344. 10.1002/(SICI)1097-0290(20000505)68:33.0.CO;2-O 10745202

[B4] Burcar B. Pasek M. Gull M. Cafferty B. J. Velasco F. Hud N. V.. (2016). Darwin's warm little pond: a one-pot reaction for prebiotic phosphorylation and the mobilization of phosphate from minerals in a urea-based solvent. Angew. Chem. Int. Ed. 55, 13249–13253. 10.1002/anie.201606239 27532228

[B5] Crick F. (1968). The origin of the genetic code. J. Mol. Biol. 38, 367–379. 10.1016/0022-2836(68)90392-6 4887876

[B6] Crick F. (1970). Central dogma of molecular biology. Nature 227, 561–563. 10.1038/227561a0 4913914

[B7] Deck C. Jauker M. Richert C. (2011). Efficient enzyme-free copying of all four nucleobases templated by immobilized RNA. Nat. Chem. 3, 603–608. 10.1038/nchem.1086 21778979

[B8] Durand E. Lecomte J. Baréa B. Dubreucq E. Lortie R. Villeneuve P. (2013). Evaluation of deep eutectic solvent-water binary mixtures for lipase-catalyzed lipophilization of phenolic acids. Green Chem. 15, 2275–2282. 10.1039/c3gc40899j

[B9] Ekland E. H. Bartel D. P. (1996). RNA-catalysed RNA polymerization using nucleoside triphosphates. Nature 382, 373–376. 10.1038/382373a0 8684470

[B10] Gabriele F. Chiarini M. Germani R. Tiecco M. Spreti N. (2019). Effect of water addition on choline chloride/glycol deep eutectic solvents: characterization of their structural and physicochemical properties. J. Mol. Liq. 291:111301 10.1016/j.molliq.2019.111301

[B11] Gállego I. Grover M. A. Hud N. V. (2015). Folding and imaging of DNA nanostructures in anhydrous and hydrated deep-eutectic solvents. Angew. Chem. Int. Ed. 54, 6765–6769. 10.1002/anie.201412354 25900011

[B12] Gao J. Wang P. Giese R. W. (2002). Xanthamide fluorescent dyes. Anal. Chem. 74, 6397–6401. 10.1021/ac020368+ 12510765

[B13] Gilbert W. (1986). Origin of life: the RNA world. Nature 319:618 10.1038/319618a0

[B14] Guajardo N. Domínguez de María H. P. Ahumada K. Schrebler R. A. Ramírez-Tagle R. Crespo F. A. (2017). Water as cosolvent: nonviscous deep eutectic solvents for efficient lipase-catalyzed esterifications. Chem. Cat Chem. 9, 1393–1396. 10.1002/cctc.201601575

[B15] Guerrier-Takada C. Gardiner K. Marsh T. Pace N. Altman S. (1983). The RNA moiety of ribonuclease P is the catalytic subunit of the enzyme. Cell 35, 849–857. 10.1016/0092-8674(83)90117-4 6197186

[B16] Gull M. Cafferty B. J. Hud N. V. Pasek M. A. (2017). Silicate-promoted phosphorylation of glycerol in non-aqueous solvents: a prebiotically plausible route to organophosphates. Life 7:29. 10.3390/life7030029 28661422 PMC5617954

[B17] Gull M. Zhou M. Fernández F. M. Pasek M. A. (2014). Prebiotic phosphate ester syntheses in a deep eutectic solvent. J. Mol. Evol. 78, 109–117. 10.1007/s00239-013-9605-9 24368625

[B18] Hammond O. S. Bowron D. T. Edler K. J. (2017). Effect of water upon deep eutectic solvent nanostructure: an unusual transition from ionic mixture to aqueous solution. Angew. Chem. Int. Ed. 56, 9782–9785. 10.1002/anie.201702486 28480595 PMC5596335

[B19] Hänle E. Richert C. (2018). Enzyme-free replication with two or four bases. Angew. Chem. Int. Ed. 57, 8911–8915. 10.1002/anie.201803074 29779237

[B20] He C. Gállego I. Laughlin B. Grover M. A. Hud N. V. (2017). A viscous solvent enables information transfer from gene-length nucleic acids in a model prebiotic replication cycle. Nat. Chem. 9, 318–324. 10.1038/nchem.2628 28338690

[B21] He C. Lozoya-Colinas A. Gállego I. Grover M. A. Hud N. V. (2019). Solvent viscosity facilitates replication and ribozyme catalysis from an RNA duplex in a model prebiotic process. Nucleic Acids Res. 47, 6569–6577. 10.1093/nar/gkz496 31170298 PMC6649698

[B22] He Y. Liu D. R. (2010). Autonomous multistep organic synthesis in a single isothermal solution mediated by a DNA walker. Nat. Nanotechnol. 5, 778–782. 10.1038/nnano.2010.190 20935654 PMC2974042

[B23] He Y. Liu D. R. (2011). A sequential strand-displacement strategy enables efficient six-step DNA-templated synthesis. J. Am. Chem. Soc. 133, 9972–9975. 10.1021/ja201361t 21657248 PMC3125949

[B24] Johnston W. K. Unrau P. J. Lawrence M. S. Glasner M. E. Bartel D. P. (2001). RNA-catalyzed RNA polymerization: accurate and general RNA-templated primer extension. Science 292, 1319–1325. 10.1126/science.1060786 11358999

[B25] Kaiser R. I. Maity S. Jones B. M. (2015). Synthesis of prebiotic glycerol in interstellar ices. Angew. Chem. Int. Ed. 54, 195–200. 10.1002/anie.201408729 25363714

[B26] Kanavarioti A. Bernasconi C. F. Doodokyan D. L. Alberas D. J. (1989). Magnesium ion catalyzed phosphorus-nitrogen bond hydrolysis in imidazolide-activated nucleotides. relevance to template-directed synthesis of polynucleotides. J. Am. Chem. Soc. 111, 7247–7257. 10.1021/ja00200a053 11542186

[B27] Kruger K. Grabowski P. J. Zaug A. J. Sands J. Gottschling D. E. Cech T. R. (1982). Self-splicing RNA: autoexcision and autocyclization of the ribosomal RNA intervening sequence of tetrahymena. Cell 31, 147–157. 10.1016/0092-8674(82)90414-7 6297745

[B28] Lannan F. M. Mamajanov I. Hud N. V. (2012). Human telomere sequence DNA in water-free and high-viscosity solvents: G-quadruplex folding governed by kramers rate theory. J. Am. Chem. Soc. 134, 15324–15330. 10.1021/ja303499m 22651378

[B29] Li L. Prywes N. Tam C. P. Oflaherty D. K. Lelyveld V. S. Izgu E. C.. (2017). Enhanced nonenzymatic RNA copying with 2-aminoimidazole activated nucleotides. J. Am. Chem. Soc. 139, 1810–1813. 10.1021/jacs.6b13148 28117989 PMC6326525

[B30] Li X. Liu D. R. (2004). DNA-templated organic synthesis: nature's strategy for controlling chemical reactivity applied to synthetic molecules. Angew. Chem. Int. Ed. 43, 4848–4870. 10.1002/anie.200400656 15372570

[B31] Liu Z. Mariani A. Wu L. Ritson D. Folli A. Murphy D.. (2018). Tuning the reactivity of nitriles using Cu(ii) catalysis-potentially prebiotic activation of nucleotides. Chem. Sci. 9, 7053–7057. 10.1039/C8SC02513D 30310625 PMC6137443

[B32] Ma C. Laaksonen A. Liu C. Lu X. Ji X. (2018). The peculiar effect of water on ionic liquids and deep eutectic solvents. Chem. Soc. Rev. 47, 8685–8720. 10.1039/C8CS00325D 30298877

[B33] Mamajanov I. Engelhart A. E. Bean H. D. Hud N. V. (2010). DNA and RNA in anhydrous media: duplex, triplex, and G-quadruplex secondary structures in a deep eutectic solvent. Angew. Chem. Int. Ed. 49, 6310–6314. 10.1002/anie.201001561 20623813

[B34] Mann S. (2012). Systems of creation: the emergence of life from nonliving matter. Acc. Chem. Res. 45, 2131–2141. 10.1021/ar200281t 22404166

[B35] Mariani A. Russell D. A. Javelle T. Sutherland J. D. (2018). A light-releasable potentially prebiotic nucleotide activating agent. J. Am. Chem. Soc. 140, 8657–8661. 10.1021/jacs.8b05189 29965757 PMC6152610

[B36] Maugeri Z. Leitner W. Domínguez De María P. (2013). Chymotrypsin-catalyzed peptide synthesis in deep eutectic solvents. Eur. J. Org. Chem. 2013, 4223–4228. 10.1002/ejoc.201300448

[B37] Meng W. Muscat R. A. McKee M. L. Milnes P. J. El-Sagheer A. H. Bath J.. (2016). An autonomous molecular assembler for programmable chemical synthesis. Nat. Chem. 8, 542–548. 10.1038/nchem.2495 27219697

[B38] Mondal D. Sharma M. Mukesh C. Gupta V. Prasad K. (2013). Improved solubility of DNA in recyclable and reusable bio-based deep eutectic solvents with long-term structural and chemical stability. Chem. Commun. 49, 9606–9608. 10.1039/c3cc45849k 24022824

[B39] Okamura H. Crisp A. Hübner S. Becker S. Rovo P. Carell T. (2019). Proto-urea-RNA (Wöhler RNA) containing unusually stable urea nucleosides. Angew. Chem. Int. Ed. 58, 18691–18696. 10.1002/anie.201911746 31573740 PMC6916321

[B40] O'Reilly R. K. Turberfield A. J. Wilks T. R. (2017). The evolution of DNA-templated synthesis as a tool for materials discovery. Acc. Chem. Res. 50, 2496–2509. 10.1021/acs.accounts.7b00280 28915003 PMC5746846

[B41] Orgel L. E. (1968). Evolution of the genetic apparatus. J. Mol. Biol. 38, 381–393. 10.1016/0022-2836(68)90393-8 5718557

[B42] Pätzold M. Siebenhaller S. Kara S. Liese A. Syldatk C. Holtmann D. (2019). Deep eutectic solvents as efficient solvents in biocatalysis. Trends Biotechnol. 37, 943–959. 10.1016/j.tibtech.2019.03.007 31000203

[B43] Paul N. Joyce G. F. (2002). A self-replicating ligase ribozyme. Proc. Natl. Acad. Sci. U.S.A. 99, 12733–12740. 10.1073/pnas.202471099 12239349 PMC130529

[B44] Smith E. L. Abbott A. P. Ryder K. S. (2014). Deep eutectic solvents (DESs) and their applications. Chem. Rev. 114, 11060–11082. 10.1021/cr300162p 25300631

[B45] Smith P. J. Arroyo C. B. Lopez Hernandez F. Goeltz J. C. (2019). Ternary deep eutectic solvent behavior of water and urea choline chloride mixtures. J. Phys. Chem. B 123, 5302–5306. 10.1021/acs.jpcb.8b12322 31242738

[B46] Steitz T. A. (2008). A structural understanding of the dynamic ribosome machine. Nat. Rev. Mol. Cell Biol. 9, 242–253. 10.1038/nrm2352 18292779

[B47] Szostak J. W. (2012). The eightfold path to non-enzymatic RNA replication. J. Syst. Chem. 3:2 10.1186/1759-2208-3-2

[B48] Tamura K. Schimmel P. (2001). Oligonucleotide-directed peptide synthesis in a ribosome- and ribozyme-free system. Proc. Natl. Acad. Sci. U.S.A. 98, 1393–1397. 10.1073/pnas.98.4.1393 11171961 PMC29267

[B49] Tamura K. Schimmel P. (2004). Chiral-selective aminoacylation of an RNA minihelix. Science 305:1253. 10.1126/science.1099141 15333830

[B50] Turk R. M. Chumachenko N. V. Yarus M. (2010). Multiple translational products from a five-nucleotide ribozyme. Proc. Natl. Acad. Sci. U.S.A. 107, 4585–4589. 10.1073/pnas.0912895107 20176971 PMC2826339

[B51] Wagle D. V. Zhao H. Baker G. A. (2014). Deep eutectic solvents: sustainable media for nanoscale and functional materials. Acc. Chem. Res. 47, 2299–2308. 10.1021/ar5000488 24892971

[B52] Xu P. Zheng G.-W. Zong M.-H. Li N. Lou W.-Y. (2017). Recent progress on deep eutectic solvents in biocatalysis. Bioresour. Bioprocess. 4, 34–52. 10.1186/s40643-017-0165-5 28794956 PMC5522511

[B53] Yarus M. (2001). On translation by RNAs alone. Cold Spring Harb. Symp. Quant. Biol. 66, 207–216. 10.1101/sqb.2001.66.207 12762023

[B54] Yonath A. (2009). Large facilities and the evolving ribosome, the cellular machine for genetic-code translation. J. R. Soc. Interface 6, S575–S585. 10.1098/rsif.2009.0167.focus 19656820 PMC2843976

[B55] Zadeh J. N. Steenberg C. D. Bois J. S. Wolfe B. R. Pierce M. B. Khan A. R.. (2009). NUPACK: analysis and design of nucleic acid systems. J. Comput. Chem. 32, 170–173. 10.1002/jcc.21596 20645303

[B56] Zhang B. Cech T. R. (1997). Peptide bond formation by *in vitro* selected ribozymes. Nature 390, 96–100. 10.1038/36375 9363898

[B57] Zhang Q. De Oliveira Vigier K. Royer S. Jérôme F. (2012). Deep eutectic solvents: syntheses, properties and applications. Chem. Soc. Rev. 41, 7108–7146. 10.1039/c2cs35178a 22806597

[B58] Zhang W. Pal A. Ricardo A. Szostak J. W. (2019). Template-directed nonenzymatic primer extension using 2-methylimidazole-activated morpholino derivatives of guanosine and cytidine. J. Am. Chem. Soc. 141, 12159–12166. 10.1021/jacs.9b06453 31298852 PMC7547883

[B59] Zhao C. Ren J. Qu X. (2013). G-quadruplexes form ultrastable parallel structures in deep eutectic solvent. Langmuir 29, 1183–1191. 10.1021/la3043186 23282194

