# Supplementary material for: Deep Eutectic Solvents as Media for the Prebiotic DNA-Templated Synthesis of Peptides
Source: Front Chem. 2020 Jan 31;8:41. doi: 10.3389/fchem.2020.00041 (PMC7005209; doi:10.3389/fchem.2020.00041)
Supplement: Supplementary file 1 [file Data_Sheet_1.pdf]

Supplementary Material

Deep eutectic solvents as media for the prebiotic DNA-templated synthesis of peptides

Samuel Núñez-Pertíñez, Thomas R. Wilks*

School of Chemistry, University of Birmingham, Edgbaston, Birmingham, B15 2TT, United Kingdom.

*** Correspondence:**
Thomas R. Wilks
T.R.Wilks@bham.ac.uk

Table S1. DNA sequences and modifications.

Product number	Sequence and modifications
S4	5'-/5ThioMC6-D/ ACG TTG CTG CAT TTT ACT CTT CTC CCC TCG GCA GCA ACG T /36-TAMTSp/-3'
S5	5'-/5Biosg/ TTT TTT TTT TTT TTT ACG TTG CTG CCG AGG GGA GAA GAG TAA AAT GCA GCA ACG T-3'
S6	5'- ACG TTG CTG CAT TTT ACT CTT CTC CCC TCG GCA GCA ACG TAA AAA AAA AAA AAA A-3'
S7	5'-/5ThioMC6-D/ GAA CTA TGT CGT TTC CGA TGG GCA CA TTT TTT T-3'
7	5'-/56-FAM/ TGT GTG CCC ATC GGA AAC GAC ATA GTT C /3AmMO/-3'
10	5'- GCC GGC CGG GCG /3AmMO/ -3'
S11	5'-/5ThioMC6-D/ ATG TAA GTA AGT CAA GTC CAG GTC GTT CAA -3'
15	5'- TTT ATT TGA ACG ACC TGG ACT TGA CTT ACT TAC AT /3AmMO/-3'
S15	5'-/5TET/ GAG GAG GGC AGC AAA CGG GAA GAG -3'
S16	5'- CTC TTC CCG TTT GCT GCC CTC CTC /3AlBkFQ/-3'

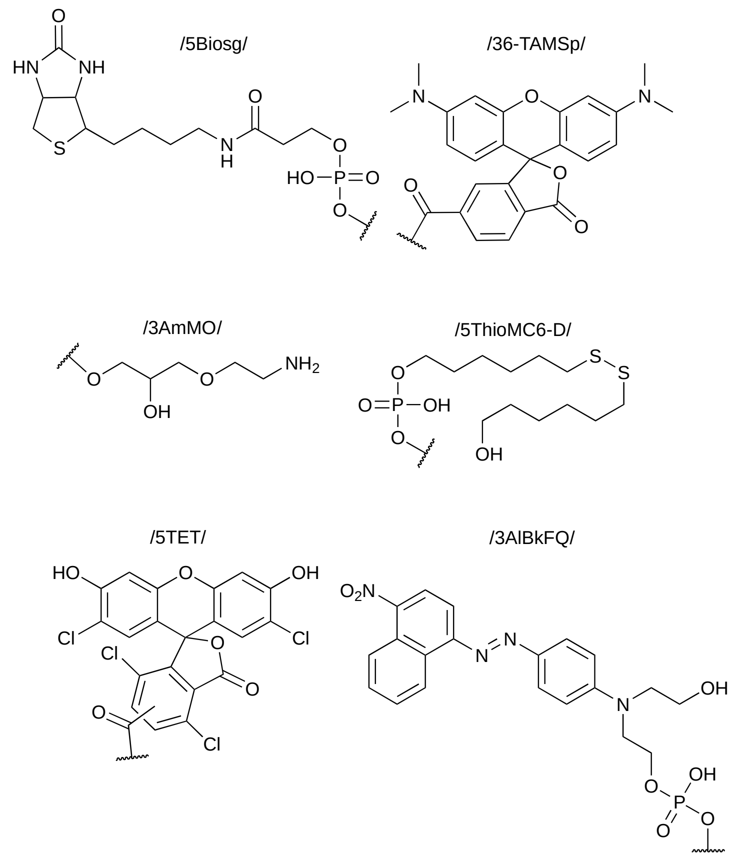

Scheme S1. Structures of DNA modifications used.

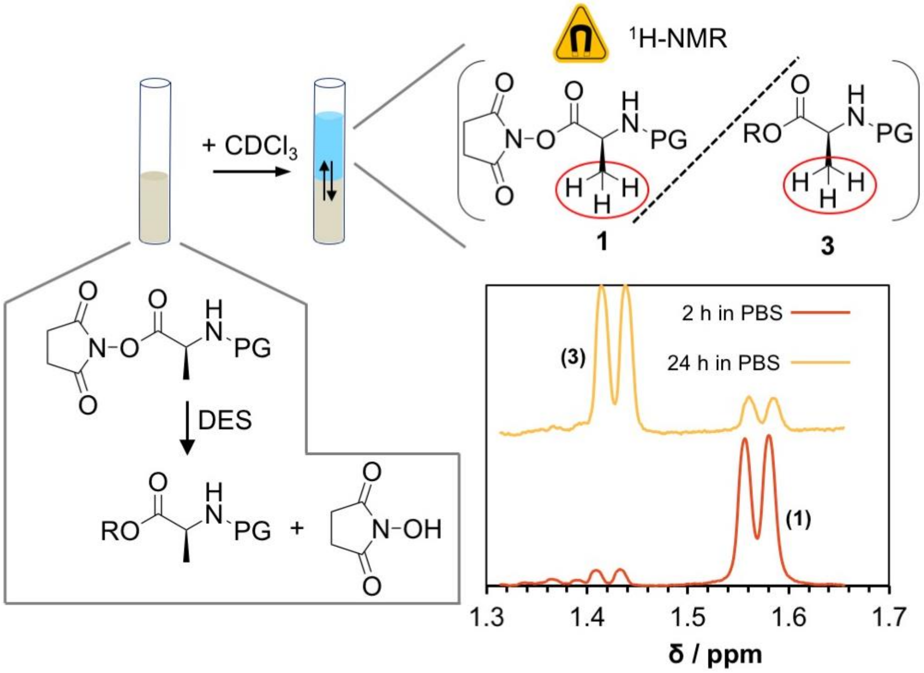

Figure S1. Schematic description of the ^{1}H-NMR spectroscopy analysis of the stability of the activated ester (1). The methylic protons produce signals at different chemical shifts (δ) before and after the solvolysis process.

Table S2. Summary of pH measurements of several glycoline/additive mixtures diluted in H_2O.

Base additive	Concentration	pH
None	-	6.8
Urea	0.3 to 3.3 M	6.6 to 6.8
Sodium acetate (NaOAc)	0.1 to 0.2 M	7.2 to 7.5
Triethylamine (Et_3N)	1 to 100 mM	7.3 to 12

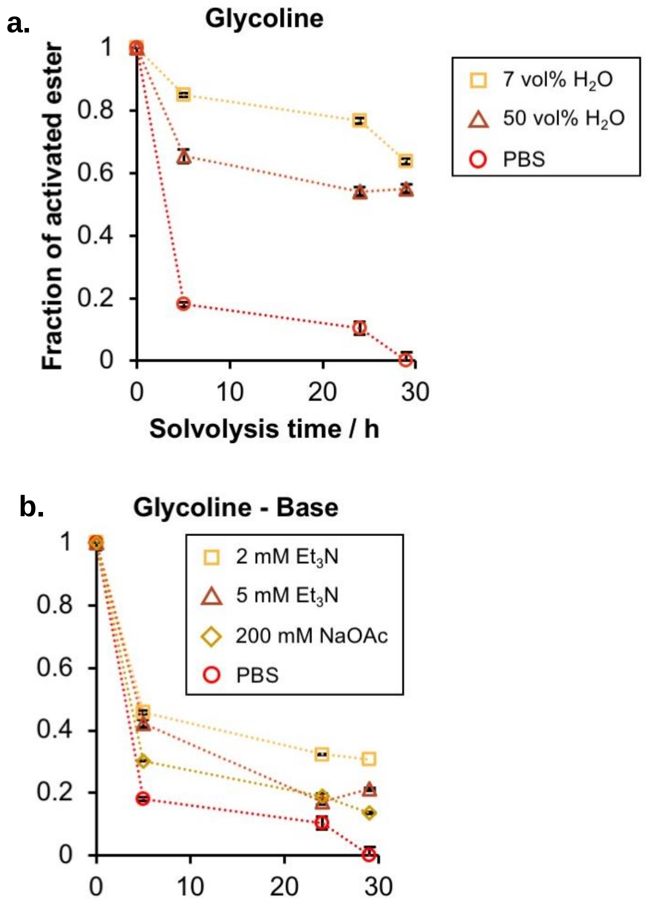

Figure S2. Results of the RP-HPLC study of activated ester stability in DES and aqueous PBS solution without (a) and with (b) the presence of basic additives.

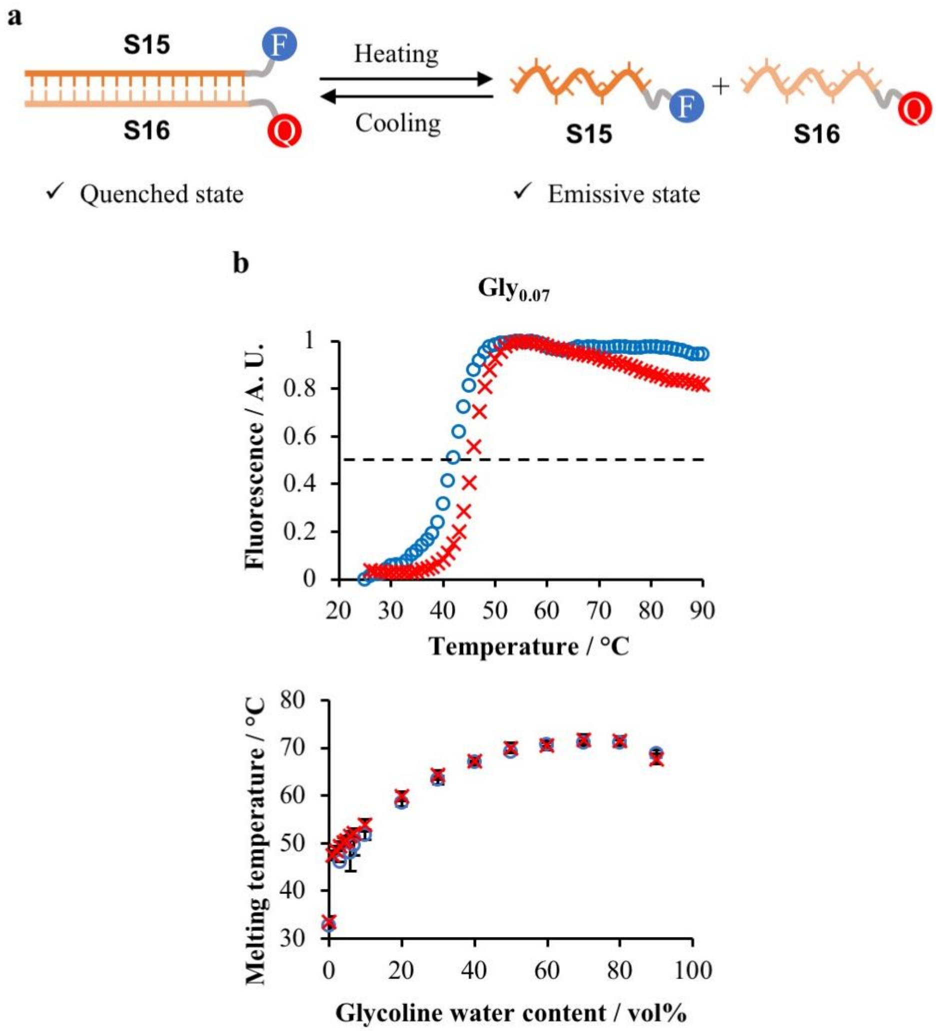

Figure S3. Thermal stability of dsDNA in DES study. a) A combination of complementary fluorophore-labelled DNA (**S15**) and quencher-labelled DNA (**S16**) were dissolved in the DES of interest. The fluorescence was recorded over a range of temperatures in a qPCR instrument. b) Melting profile of dsDNA in glycoline with 7 vol% H_2O ($Gly_{0.07}$). Evolution of the T_m as function of the H_2O content in glycoline. The experiments were run in triplicate and the error was determined as the standard deviation.

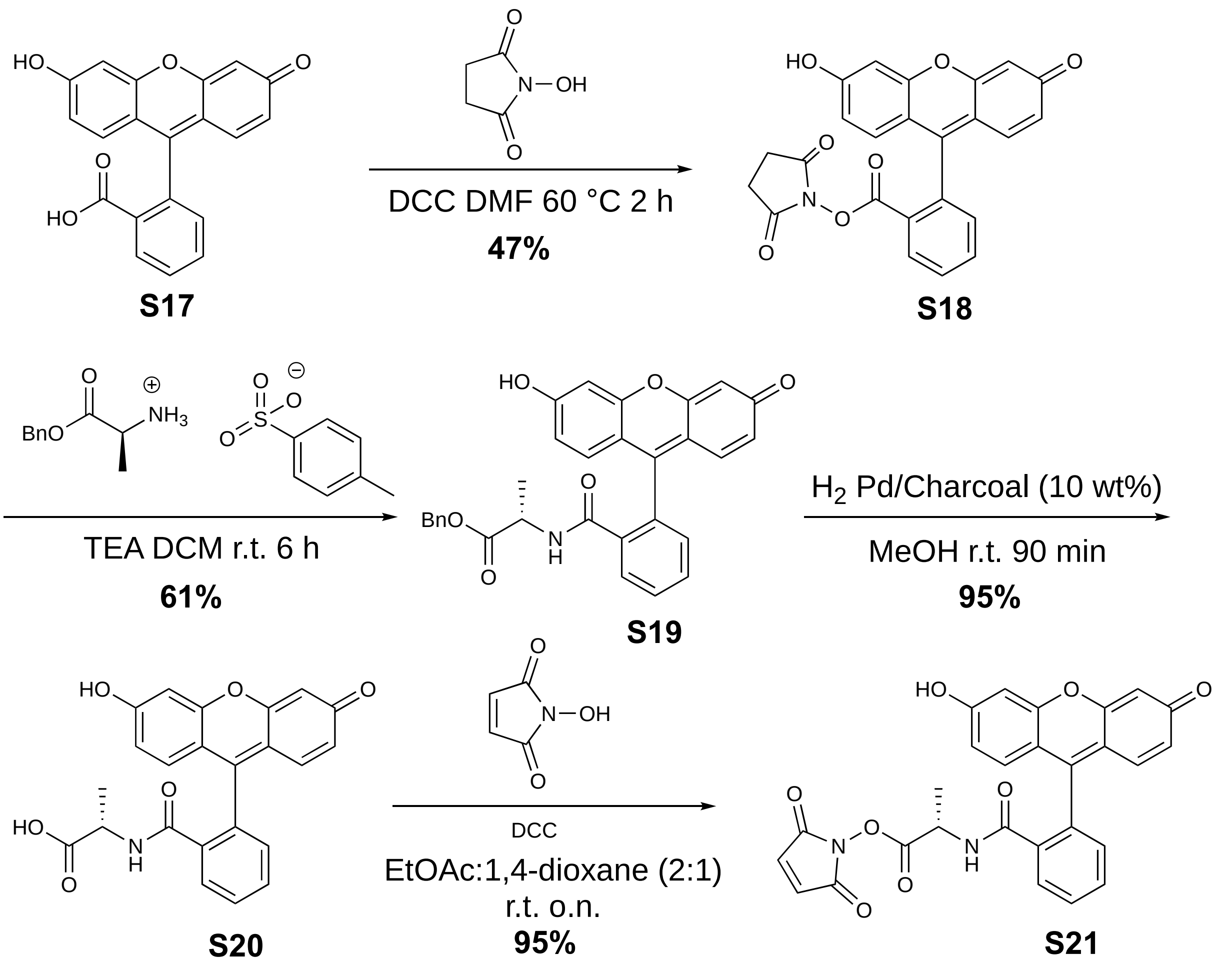

Scheme S2. Synthesis of the the FAM-labelled NHS-activated ester of alanine, **S21**.

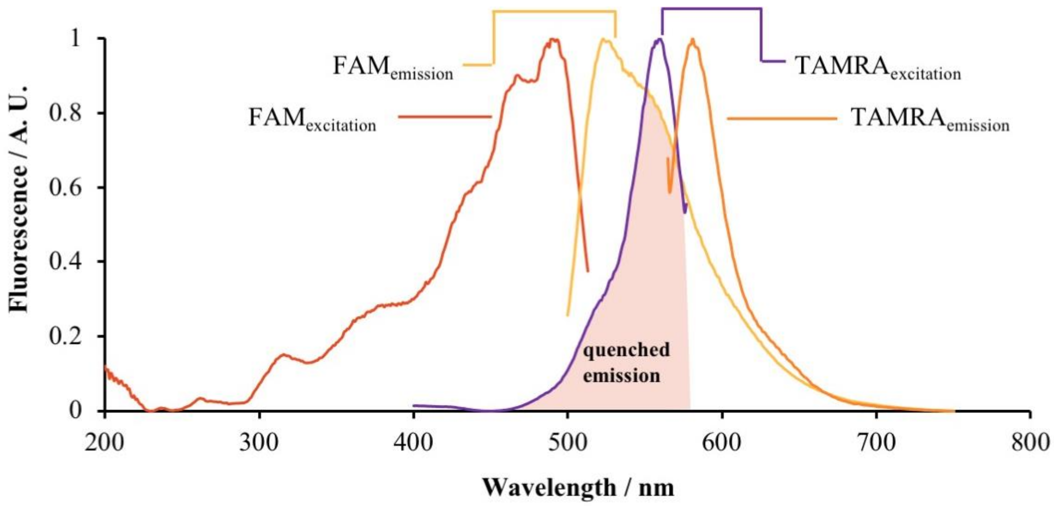

Figure S4. Fluorescence excitation and emission spectra in Gly$_{0.07}$ of **S21** and tetramethyl rhodamine (TAMRA)-labelled DNA **S4**. The overlap between the FAM fluorophore emission and the TAMRA excitation shows that TAMRA is a suitable quencher.

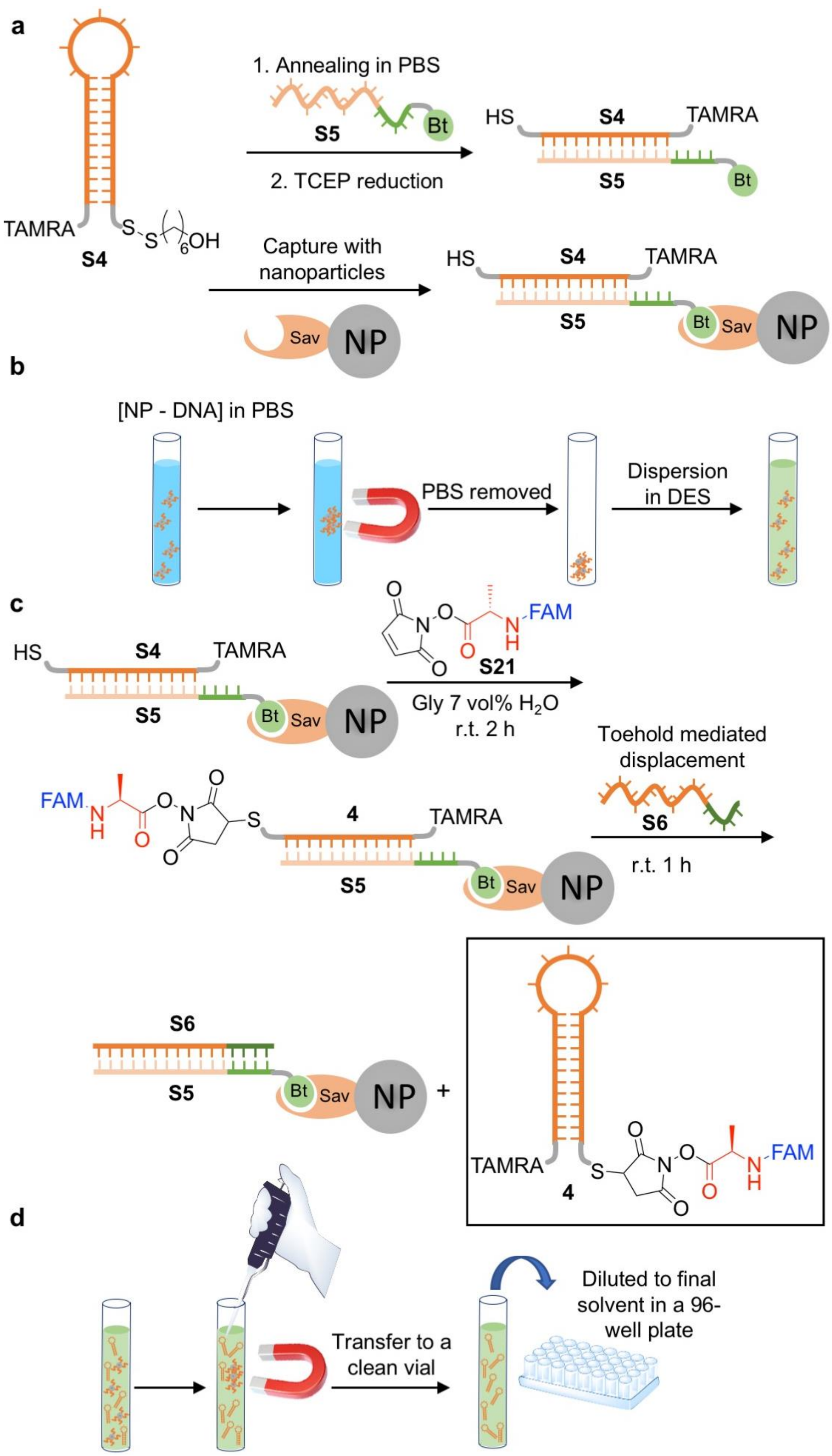

Figure S5. Schematic description of the preparation of the labelled hairpin **4**.

Table S3. Additives assayed to improve the conversion of DTS in DES

Solvent	Additives	Result
Glycoline	7 vol% H_2O	No product detected
Glycoline	20 vol% H_2O	No product detected
Glycoline	40 vol% H_2O	No product detected
Glycoline	7 vol% H_2O / 500 mM NaOAc	Low conversion (< 5%)
Glycoline	7 vol% H_2O / 100 mM TEA	Low conversion (< 5%)
Glycoline	7 vol% H_2O / 5 mM TEA	No product detected
